# Comparative Outcomes of Ultrasound-Assisted Thrombolysis and Mechanical Thrombectomy in Intermediate-High-Risk Pulmonary Embolism

**DOI:** 10.3390/jcm15114023

**Published:** 2026-05-22

**Authors:** Claudia Colombo, Marco Zuin, Filippo Russo, Mario Iannaccone, Marco Solcia, Francesco Musca, Ilaria Emanuela Bossi, Andrea Cesari, Elena Gualini, Federica Fumarola, Alberto Balderi, Francesca Giordana, Andrea Discalzi, Lorenzo Tua, Stefano Buratti, Ruggero Vercelli, Lorenzo Cianfanelli, Marianna Adamo, Alaide Chieffo, Giacomo Bocuzzi, Matteo Montorfano, Fabrizio Oliva, Alice Sacco

**Affiliations:** 1 Cardiology 1, De Gasperis Cardio Center, ASST Grande Ospedale Metropolitano Niguarda, 20162 Milan, Italy; claudia.colombo@ospedaleniguarda.it (C.C.); andrea.cesari@ospedaleniguarda.it (A.C.); elena.gualini@ospedaleniguarda.it (E.G.); fabrizio.oliva@ospedaleniguarda.it (F.O.); 2 Department of Translational Medicine, University of Ferrara, 44100 Ferrara, Italy; marco.zuin@aulss6.veneto.it; 3 Department of Cardio-Thoraco-Vascular Sciences and Public Health, University of Padova, 35128 Padua, Italy; 4 Department of Cardiology, Madre Teresa di Calcutta Hospital, AULSS 6, South Padova Hospitals, 35043 Schiavonia, Italy; 5 Interventional Cardiology Unit, IRCCS San Raffaele Scientific Institute, 20132 Milan, Italy; russo.filippo@hsr.it (F.R.); chieffo.alaide@hsr.it (A.C.); montorfano.matteo@hsr.it (M.M.); 6 Cardiology Unit, San Giovanni Bosco, ASL Città di Torino, 10128 Turin, Italy; mario.iannaccone@hotmail.it (M.I.); giacomoboccuzzi@aslcittaditorino.it (G.B.); 7 Interventional Radiology Unit, ASST Grande Ospedale Metropolitano Niguarda, 20162 Milan, Italy; marco.solcia@ospedaleniguarda.it; 8 Cardiology 4, De Gasperis Cardio Center, ASST Grande Ospedale Metropolitano Niguarda, 20162 Milan, Italy; francesco.musca@ospedaleniguarda.it; 9 Department of Emergency Medicine, ASST Grande Ospedale Metropolitano Niguarda, 20162 Milan, Italy; ilaria.bossi@unimi.it; 10 Interventional Radiology Unit, San Giovanni Bosco, ASL Città di Torino, 10128 Turin, Italy; federica.fumarola.gm@gmail.com; 11 Unit of Interventional Radiology, Department of Radiology, A.O. S. Croce e Carle, 12100 Cuneo, Italy; balderi.a@ospedale.cuneo.it; 12 Division of Cardiology, A.O. S. Croce e Carle, 12100 Cuneo, Italy; francy.giordana@gmail.com; 13 Radiology Unit, Department of Surgical Sciences, University of Torino, 12100 Turin, Italy; andreadiscalzi@gmail.com; 14 Interventional Cardiology Unit, ASST Santi Paolo e Carlo, San Carlo Borromeo Hospital, 20142 Milan, Italy; lorenzo.tua1@gmail.com; 15 Cardiology Division, ASST Santi Paolo and Carlo, San Paolo Hospital, 20142 Milan, Italy; stefano.buratti@asst-santipaolocarlo.it; 16 Interventional Radiology Unit, ASST Santi Paolo Carlo, 20142 Milan, Italy; ruggero.vercelli@asst-santipaolocarlo.it; 17 Intensive Care Unit, IRCCS San Raffaele Scientific Institute, 20132 Milan, Italy; cianfanelli.lorenzo@hsr.it; 18 Institute of Cardiology, ASST Spedali Civili di Brescia, 25123 Brescia, Italy; mariannaadamo@hotmail.com; 19 School of Medicine, Vita-Salute San Raffaele University, 20132 Milan, Italy

**Keywords:** catheter-directed therapies (CDT), ultrasound-assisted catheter-directed thrombolysis (USAT), mechanical thrombectomy (MT)

## Abstract

**Background/Objectives**: Ultrasound-assisted catheter-directed thrombolysis (USAT) and mechanical thrombectomy (MT) are increasingly used for intermediate-high-risk pulmonary embolism (PE), but real-world comparisons are scarce. We compare 30-day all-cause, cardiovascular, non-cardiovascular, PE-related, and major bleeding events between intermediate-high-risk patients with acute PE treated with USAT or MT. **Methods**: We analyzed 286 patients with acute intermediate-high-risk PE enrolled in the multicenter USAT IH-PE registry (March 2019–October 2025). Patients underwent USAT (EKOS™, Boston Scientific, Marlborough, MA, USA) or MT (FlowTriever, Inari Medical, Irvine, CA, USA or Indigo, Penumbra, Inc., Alameda, CA, USA) during index hospitalization. Primary endpoints were 30-day all-cause, cardiovascular, and PE-related mortality. Propensity score matching (1:1) balanced baseline characteristics. Kaplan–Meier analyses with log-rank testing assessed time-to-event outcomes. **Results**: After matching (69 patients per group), baseline clinical and hemodynamic variables were well balanced. Both USAT and MT significantly improved RV/LV ratio, tricuspid annular plane systolic excursion (TAPSE), and PASP (all *p* < 0.001). Thirty-day all-cause mortality was similar between USAT and MT (13.0% vs. 11.5%; *p* = 0.78), with no differences in cardiovascular or PE-related mortality (8.6% vs. 1.4%, *p* = 0.58 and 5.7% vs. 1.4%, *p* = 0.17, respectively). Major bleeding was infrequent and observed only in the USAT group (4.3%). **Conclusions**: In this real-world multicenter cohort, USAT and MT showed comparable short-term mortality, safety, and echocardiographic recovery, supporting individualized catheter-based reperfusion strategies for intermediate-high-risk PE.

## 1. Introduction

Catheter-directed reperfusion therapies (CDTs) have recently emerged as appealing alternatives to anticoagulation alone in patients with intermediate-high-risk acute pulmonary embolism (PE) who develop clinical deterioration [1,2]. These interventions aim to rapidly reduce thrombus burden and alleviate right ventricular (RV) pressure overload, while potentially limiting bleeding complications associated with systemic thrombolysis [1,3]. In contemporary clinical practice, the selection of catheter-directed reperfusion strategies is generally based on an individualized assessment of bleeding risk, thrombus burden, and hemodynamic status [1,2,3]. Catheter-directed lytic therapy (CDL), including ultrasound-assisted systems, is typically preferred in patients with significant RV dysfunction and low-to-intermediate bleeding risk, in whom rapid pharmacological thrombus reduction is desired while minimizing systemic thrombolytic exposure. In contrast, mechanical thrombectomy (MT) is more frequently considered in patients with higher bleeding risk, contraindications to thrombolysis, or those requiring immediate clot extraction due to more severe hemodynamic compromise or extensive central thrombus burden. Despite these general principles, real-world decision-making remains highly heterogeneous and is largely influenced by institutional protocols, operator experience, and multidisciplinary Pulmonary Embolism Response Team (PERT) evaluation. Importantly, robust randomized evidence defining clear superiority or patient-specific indications for one strategy over another is still lacking, leaving substantial uncertainty regarding optimal device selection in routine clinical practice. However, current clinical evidence supporting CDTs has largely relied on surrogate endpoints, such as reductions in pulmonary artery systolic pressure (PASP), improvements in RV/left ventricular (LV) ratio, and echocardiographic markers of RV function [1,2]. Although these parameters are pathophysiologically meaningful and reflect early hemodynamic improvement, their translation into clinically relevant benefits remains uncertain. In contrast, hard outcomes such as all-cause mortality, cardiovascular (CV) mortality, and PE-related mortality have been less consistently evaluated across studies [4,5,6]. Furthermore, comparative data across different CDT modalities remain limited, as most randomized and prospective studies have adopted single-arm designs or evaluated individual techniques in isolation rather than direct head-to-head comparisons [7]. As a result, the current evidence base remains fragmented and provides limited guidance for clinicians when selecting the most appropriate reperfusion strategy in daily practice [3].

In this context, real-world comparative analyses are particularly valuable, as they reflect contemporary management strategies and include broader and more heterogeneous patient populations than those typically enrolled in randomized controlled trials. Accordingly, the objective of this study was to compare the effectiveness and safety of USAT-CDT versus MT in a multicenter real-world registry of patients with intermediate-high-risk acute PE. Specifically, we aimed to evaluate whether the two strategies differ in terms of short-term clinically meaningful outcomes, including 30-day all-cause mortality, cardiovascular mortality, PE-related mortality, and major bleeding.

We hypothesized that, despite differences in mechanism of action and patient selection, USAT and MT would be associated with comparable improvements in right ventricular function and similar short-term clinical outcomes in a contemporary real-world setting.

## 2. Materials and Methods

### 2.1. Study Population: USAT-IH

As previously described, the USAT IH-PE registry is a multicenter, prospective, observational study enrolling consecutive patients hospitalized at eight Italian tertiary-care centers with established expertise in PE (NCT06143969). A full list of enrolling centers is shown in Supplementary Table S1.

For the present analysis, we included patients with intermediate-high-risk acute PE admitted between March 2019 and October 2025 who underwent catheter-based reperfusion during the index hospitalization. Reperfusion strategies comprised USAT-CDT using the EKOS™ system (Boston Scientific, Marlborough, MA, USA) or MT using commercially available devices (FlowTriever^®^, Inari Medical, Irvine, CA, USA; Indigo^®^, Penumbra, Inc., Alameda, CA, USA). Treatment selection was guided by multidisciplinary Pulmonary Embolism Response Teams (PERTs) according to local protocols. PE risk stratification was performed in accordance with the 2019 European Society of Cardiology (ESC) guidelines [1].

Inclusion criteria were (i) age ≥ 18 years, (ii) confirmed diagnosis of intermediate-high-risk PE at contrast computed tomography angiography (CTPA), with thrombus involving at least one main or proximal lower pulmonary artery, (iii) with or without deep vein thrombosis (DVT), (iv) symptom onset within 14 days prior to admission, and (v) availability of complete clinical, imaging, and follow-up data required for risk stratification and outcome assessment. Patients were excluded if they were (i) unable to provide informed consent, (ii) were pregnant, (iii) had missing key variables required for the 2019 ESC risk stratification or unavailable follow-up data; (iv) had received fibrinolytic therapy within the previous 4 days, or (v) had conditions conferring a high bleeding or procedural risk, including known coagulation disorders, platelet count < 100,000/µL, gastrointestinal bleedings within the preceding 3 months, (vi) an active malignancy with an expected survival < 6 months, or (vii) advanced chronic kidney disease (defined as an estimated glomerular filtration rate < 30 mL/min/1.73 m^2^ or chronic dialysis). The application of exclusion criteria was left to the discretion of the treating physicians according to local protocols and multidisciplinary team evaluation.

The registry was conducted in accordance with the Declaration of Helsinki, and the study protocol was approved by the ethics committee of the coordinating center as well as by the institutional review boards of all participating centers. Written informed consent was obtained from all patients prior to inclusion.

### 2.2. Definitions

Baseline demographics, clinical characteristics, transthoracic echocardiography (TTE) parameters, and 30-day clinical outcomes were systematically collected. TTE was performed at admission for risk stratification. Measurements were averaged over three cardiac cycles in patients with sinus rhythm and over five cycles in those with atrial fibrillation. RV dysfunction was defined as an RV/left ventricular (LV) end-diastolic diameter ratio > 1 in the apical 4-chamber view. RV systolic dysfunction was defined as a tricuspid annular plane systolic excursion (TAPSE) < 17 mm, in accordance with current echocardiographic recommendations. Pulmonary artery systolic pressure (PASP) was calculated from the peak tricuspid regurgitant jet velocity using the modified Bernoulli equation, with estimated right atrial pressure added [8]. Pulmonary embolism clot burden and anatomical distribution were assessed by CTPA according to local protocols. Cardiac troponins as well as serum lactate levels were measured according to local standards.

### 2.3. Ultrasound-Assisted Catheter-Directed Thrombolysis

Echo-guided central venous access was obtained via either the jugular or femoral vein, according to patient-specific anatomy and operator preference. No pulmonary angiography was performed, and the EKOS™ system was deployed under fluoroscopic guidance based on the angio-CT scan thrombus distribution. In cases of unilateral PE involving a main or proximal lobar pulmonary artery, a single catheter was positioned within the affected vessel. For bilateral embolism, affecting the main or proximal lobar pulmonary arteries, two catheters were inserted, one in each involved artery. Alteplase was administered as a continuous infusion of 10 mg per catheter over a 10 h period (1 mg/h). Concurrently, intravenous unfractionated heparin (UFH) was initiated and adjusted to achieve a target activated partial thromboplastin time of 50–70 s. At the completion of the 10 h infusion, thrombolytic therapy was discontinued, the EKOS™ catheters were removed, and subsequently anticoagulation was continued [3,4,5].

### 2.4. Mechanical Thrombectomy

Mechanical Thrombectomy could be performed using either the FlowTriever^®^ retrieval/aspiration system (16 Fr, 20 Fr, and 24 Fr) [9,10] or the Indigo^®^ aspiration system (Penumbra Inc., CA, USA) (12 Fr and 16 Fr) at the operator’s discretion and based on the local availability of the device [10]. Specifically, this latter system was also equipped with a computer-aided automatic valve control (Lightning system, Penumbra Inc., CA, USA), facilitating thrombus removal [10].

### 2.5. Outcomes

The primary outcomes included 30-day all-cause mortality, cardiovascular mortality, and PE-related mortality, analyzed within a hierarchical mortality outcome framework. The rationale for this structured approach was to provide a comprehensive assessment of short-term clinically meaningful outcomes while also allowing mechanistic attribution of death in a condition where early mortality is often multifactorial and variably classified across studies. In intermediate-high-risk pulmonary embolism, death may result from direct PE-related hemodynamic collapse, secondary cardiovascular complications (e.g., right ventricular failure or arrhythmias), or non-cardiovascular causes, frequently within overlapping clinical trajectories. Therefore, analyzing mortality through complementary but distinct adjudication categories enables both a global assessment of treatment-associated outcomes (all-cause mortality) and a more granular evaluation of cause-specific mechanisms potentially influenced by reperfusion strategy. These outcomes were not intended to represent a traditional statistical composite endpoint, but rather a structured hierarchy of clinically relevant mortality categories aimed at providing a more detailed characterization of short-term outcomes in a real-world population. Secondary endpoints included the occurrence of in-hospital bleeding events, which were adjudicated according to the Bleeding Academic Research Consortium (BARC) criteria [11,12,13,14].

### 2.6. Statistical Analysis

Continuous variables are expressed as mean ± standard deviation and were compared using Student’s *t* test or the Mann–Whitney U test, as appropriate, according to data distribution. Categorical variables are presented as counts and percentages and were compared using the chi-square test or Fisher’s exact test, as appropriate. Within-group changes in echocardiographic parameters before and after the procedure were assessed using paired *t* tests or Wilcoxon signed-rank tests, as applicable.

Given the non-randomized nature of treatment allocation, propensity score matching (PSM) was performed to minimize baseline differences between patients treated with USAT and MT. Propensity scores were estimated using a multivariable logistic regression model including clinically relevant baseline covariates potentially influencing both treatment selection and outcomes. These variables comprised demographic characteristics (age, sex, body mass index), hemodynamic parameters at presentation (systolic and diastolic blood pressure, heart rate), cardiovascular risk factors and comorbidities (systemic hypertension, diabetes mellitus, chronic kidney disease, chronic obstructive pulmonary disease, active or previous smoking status, cancer, heart failure, coronary artery disease, prior stroke, previous deep vein thrombosis, and prior pulmonary embolism), echocardiographic markers of right ventricular dysfunction (RV/LV end-diastolic diameter ratio, TAPSE, pulmonary artery systolic pressure, and presence of RV hypokinesia), and computed tomography pulmonary angiography (CTPA)-derived measures of embolic burden and distribution (bilateral main, bilateral lobar, unilateral main, or unilateral lobar involvement). Baseline anticoagulant therapy (direct oral anticoagulants, low-molecular-weight heparin, or vitamin K antagonists) was also included in the model. Where available, baseline serum lactate was considered in sensitivity-adjusted analyses as an additional marker of occult hypoperfusion. Patients were matched 1:1 using nearest-neighbor matching without replacement and a predefined caliper width. Covariate balance after matching was assessed using standardized mean differences, with values < 0.10 indicating adequate balance. Time-to-event analyses were then performed in the matched cohort, and patients were censored at 30 days or at last available follow-up. This follow-up period was selected because it represents the phase of highest clinical risk in intermediate-high-risk PE and in PE more generally, during which most procedure- and treatment-related benefits and complications are expected to occur. Moreover, this time horizon is also consistent with international guidelines and validated risk stratification models, which predominantly rely on in-hospital or 30-day mortality as the reference standard for early prognosis and treatment evaluation in acute PE. In addition, complete and standardized long-term follow-up beyond 30 days was not uniformly available across all participating centers, and the 30-day window ensured data completeness while minimizing potential attrition bias in this multicenter real-world registry. For the primary analysis, MT was considered as a single therapeutic category, encompassing FlowTriever^®^ and Indigo^®^ systems. To evaluate potential heterogeneity, sensitivity analyses were conducted according to device type, which showed consistent clinical and echocardiographic outcomes across both systems, without statistically significant differences. A two-tailed *p* value < 0.05 was considered statistically significant. All analyses were conducted using SPSS version 27.0 (IBM Corp, Armonk, NY, USA).

## 3. Results

### 3.1. Baseline Characteristics in the Overall Cohort

**Figure 1:**
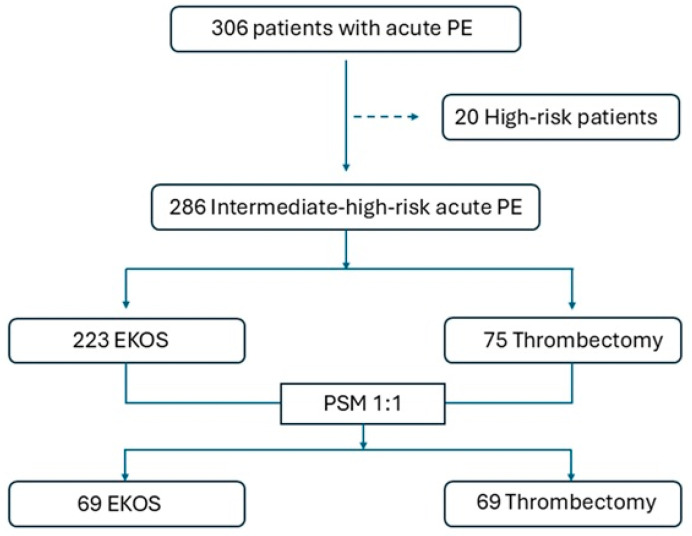
Study flowchart. PE: pulmonary embolism. PSM: propensity score matching.

Over the study period, 306 patients with acute PE were enrolled in the registry. Among them, 20 high-risk patients were excluded, leaving 286 intermediate-high-risk patients. Of these, 211 underwent USAT with EKOS, while 75 underwent MT with the FlowTriever or Penumbra (Table 1 and Figure 1). Mean age was similar between groups (64.7 ± 14.5 vs. 64.4 ± 17.8 years; *p* = 0.88), as was the proportion of female patients (52.6% vs. 52.0%; *p* = 0.92). Body mass index did not differ significantly between groups (29.6 ± 5.6 vs. 29.8 ± 5.9 kg/m^2^; *p* = 0.78). Within the mechanical thrombectomy cohort (n = 69), 45% were treated with the FlowTriever^®^ system and 55% with the Indigo^®^ aspiration system.

At presentation, patients treated with USAT had higher systolic blood pressure (SBP) and diastolic blood pressure (DBP) compared with those undergoing MT (SBP: 131.7 ± 11.4 vs. 125.9 ± 15.0 mmHg, *p* < 0.001; DBP: 80.2 ± 13.3 vs. 75.2 ± 14.4 mmHg, *p* = 0.007), whereas heart rate was comparable. Cardiovascular risk profiles differed between groups: MT patients more frequently suffered from chronic kidney disease (29.3% vs. 9.9%; *p* < 0.001), diabetes mellitus (41.3% vs. 11.8%; *p* < 0.001), and chronic obstructive pulmonary disease (21.3% vs. 4.3%; *p* < 0.001). Prior smoking was also more prevalent in the MT group (33.3% vs. 10.9%; *p* = 0.009).

Baseline echocardiography demonstrated more severe RV involvement in the MT group, with a higher RV/LV ratio (1.4 ± 0.2 vs. 1.2 ± 0.1; *p* < 0.001), although RV hypokinesia was more frequently reported in the USAT group (76.3% vs. 42.6%; *p* < 0.001). TAPSE was modestly higher in USAT patients (15.8 ± 4.7 vs. 14.7 ± 15.8; *p* = 0.04), whereas PASP was similar between groups (51 ± 16.2 vs. 51.4 ± 12.4; *p* = 0.84). PE anatomical distribution on CTPA and concomitant anticoagulant therapy did not differ significantly. A sensitivity analysis exploring baseline clinical, echocardiographic, and radiological characteristics among patients treated with FlowTriever^®^ or Indigo^®^ revealed no differences among groups (Supplementary Table S2).

### 3.2. Propensity Score–Matched Cohort

After 1:1 propensity score matching, 138 patients (69 per group) were included in the matched analysis (Table 2). Baseline demographic variables, cardiovascular risk factors, comorbidities, and prior thromboembolic history were well balanced between the USAT and MT groups. Hemodynamic parameters, echocardiographic indices, PE burden, and background anticoagulant treatment showed no statistically significant differences, indicating adequate covariate balance. Moreover, 31 matched pairs were obtained (50% in the FlowTriever^®^ group and 50% in the Indigo^®^ group, respectively), with well-balanced covariates.

### 3.3. Echocardiographic Changes After Intervention

In both the USAT and MT cohorts, significant post-procedural improvements were observed across all echocardiographic parameters (Supplementary Tables S3 and S4). In the USAT cohort, the RV/LV ratio decreased from 1.2 ± 0.3 to 0.79 ± 0.1, TAPSE increased from 15.2 ± 2.0 to 22.6 ± 0.3 mm, and PASP declined from 49.3 ± 11.3 to 30.7 ± 3.2 mmHg (all *p* < 0.001). Similarly, in the MT cohort, the RV/LV ratio declined from 1.3 ± 0.4 to 0.90 ± 0.2, TAPSE increased from 14.3 ± 3.1 to 18.9 ± 0.8 mm, and PASP decreased from 50.3 ± 10.6 to 33.6 ± 4.2 mmHg (all *p* < 0.001). Between-group comparisons of delta echocardiographic parameters confirmed no significant differences in the magnitude of right ventricular functional recovery between USAT and MT (Table 3 and Supplementary Table S5).

### 3.4. Clinical and Time-to-Event Outcomes

In the matched cohort, Kaplan–Meier analyses showed similar all-cause mortality at 30 days between USAT and MT patients (13.0% vs. 11.5%; *p* = 0.78). Rates of cardiovascular death, non-CV death, and PE-related mortality did not differ significantly between treatment strategies as well (Table 2).

**Figure 2:**
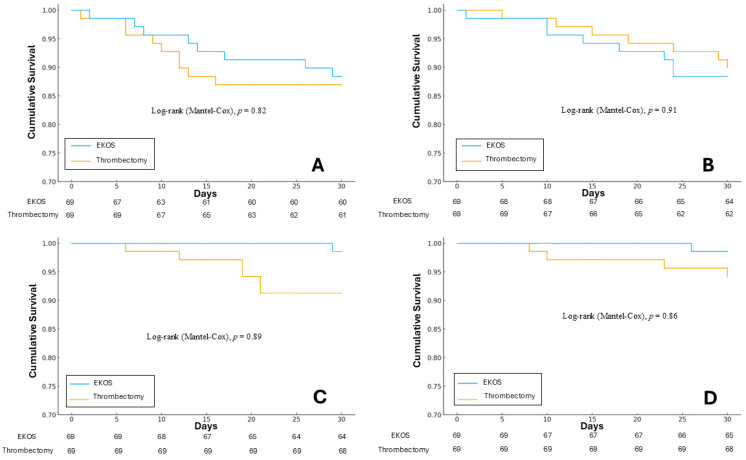
Kaplan–Meier curves for 30-day all-cause mortality (**A**), cardiovascular mortality (**B**), non-cardiovascular mortality (**C**), and pulmonary embolism-related mortality (**D**), stratified by catheter-directed reperfusion strategy.

All-cause mortality curves were largely overlapping throughout follow-up (log-rank Mantel–Cox, *p* = 0.82) (Figure 2A). Non-CV death showed comparable trajectories between treatment strategies, with a small and gradual decline in survival over time (Figure 2B) (log-rank Mantel–Cox, *p* = 0.91). Similarly, CV mortality remained low in both groups, with no early separation of the curves (Figure 2C) (log-rank Mantel–Cox, *p* = 0.89). PE–related mortality was infrequent, with high event-free survival in both groups and no apparent divergence between USAT and MT (Figure 2D) (log-rank Mantel–Cox, *p* = 0.86) (Supplementary Table S6).

### 3.5. Bleeding Events

Bleeding complications assessed according to the BARC classification are shown in Supplementary Table S5. No bleeding events occurred in 68.1% of USAT patients and 81.1% of the MT group (*p* = 0.08). Major bleeding events (BARC ≥ 3) were infrequent in both groups and occurred only in the USAT cohort, with no statistically significant difference between groups (Supplementary Table S7).

### 3.6. Sensitivity Analysis for Thrombectomy Devices

No significant differences were observed in 30-day mortality, PE-related mortality, or bleeding events between devices for MT. Moreover, both systems were associated with significant and comparable improvements in RV/LV ratio, TAPSE, and PASP (all *p* < 0.001).

## 4. Discussion

### 4.1. Main Findings

In this real-world, multicenter registry of patients with intermediate-high-risk acute PE treated with catheter-based reperfusion systems, both USAT and MT were associated with substantial improvements in hemodynamic status, RV function, and short-term clinical outcomes. Importantly, no significant differences were observed between treatment strategies in terms of CV, PE-related, and all-cause mortality at 30 days. Additionally, patients treated with FlowTriever^®^ and Indigo^®^ systems experienced comparable short-term outcomes.

In the unmatched cohort, mechanical thrombectomy patients showed a higher comorbidity burden and more severe baseline RV dysfunction, likely reflecting real-world, PERT-driven selection rather than random variation. MT was more often used in patients with higher bleeding risk or greater systemic comorbidities, whereas USAT was preferentially selected for patients considered suitable for thrombolysis, underscoring inherent indication bias in observational comparisons. Although no significant differences in 30-day outcomes were observed after propensity score matching, these findings should not be interpreted as evidence of equivalence. The study may be underpowered for infrequent endpoints, and residual confounding cannot be excluded; therefore, results should be considered hypothesis-generating within the limits of an observational design.

Both percutaneous reperfusion strategies were associated with significant and consistent improvements in key echocardiographic markers of RV pressure overload, including RV/LV ratio, TAPSE, and PAPS [15,16,17]. While these parameters represent surrogate markers of clinical improvement, their concordant and robust changes underscore the effectiveness of catheter-based reperfusion in rapidly reversing acute RVD in intermediate-high-risk PE. Importantly, our study extends beyond surrogate endpoints by demonstrating comparable rates of hard clinical outcomes, including CV, PE-related, and all-cause mortality, thereby reinforcing the clinical relevance of the observed echocardiographic recovery [17,18].

Moreover, patients treated with FlowTriever^®^ and Indigo^®^ systems experienced comparable short-term outcomes, including all-cause, CV, non-CV, and PE-related mortality, suggesting that both catheter-based thrombectomy devices are similarly effective and safe in the management of intermediate-high-risk PE. Emerging evidence suggests that a substantial proportion of intermediate-high risk PE patients already harbor signs of occult hypoperfusion at presentation despite maintaining normal blood pressure [19,20]. Plasma lactate, a sensitive marker of tissue oxygen supply-demand imbalance, has been shown to carry independent prognostic value in normotensive PE patients beyond conventional markers of right ventricular dysfunction [21].

In the present cohort, lactate levels were higher in the MT group, which received the more immediate reperfusion strategy. This finding is consistent with a real-world tendency to select MT for patients perceived to be at greater hemodynamic vulnerability, those in whom the urgency of clot removal outweighs the time required for thrombolytic infusion. Present results are also consistent with prior observational studies and meta-analyses reporting similar short-term mortality and hemodynamic outcomes between USAT and MT, with differences primarily related to procedural characteristics, length of hospital stay, or bleeding profiles rather than major clinical endpoints [22,23,24]. In this context, the low and non-significantly different rates of major bleeding observed in our cohort are reassuring. Discrepancies with larger registries such as REAL-PE [22], REAL-PE II [24], and meta-analysis [25], which have reported differing bleeding risks, may reflect variations in patient selection, sample size, and study design.

### 4.2. Comparison with Randomized and Prospective Evidence

Our results are broadly consistent with previous randomized and prospective studies evaluating CDTs in intermediate-high-risk patients with acute PE, such as the PEERLESS II trial [23], which currently represents the largest contemporary randomized study comparing catheter-based reperfusion strategies in acute PE. While PEERLESS II employed a randomized design and a hierarchical composite endpoint incorporating clinical deterioration and procedural outcomes, our analysis reflects real-world practice and focuses on hard clinical endpoints, including short-term mortality. Despite these methodological differences, both studies consistently demonstrate significant hemodynamic improvement following catheter-based interventions and do not identify a clear superiority of one strategy over another in terms of major clinical outcomes. Importantly, our registry extends these findings to a broader and more comorbid population, also exploring hard outcomes, supporting the external validity and generalizability of catheter-based reperfusion strategies in routine clinical settings. The parallel improvement in RV indices observed with both treatment modalities supports the concept that early reduction in RV afterload, rather than the specific device or mechanism of thrombus removal, is the principal driver of early clinical stabilization in intermediate-high-risk PE. This interpretation is further supported by emerging prospective data, including the PEERLESS trial [23], which suggested procedural advantages of large bore thrombectomy in selected patients without demonstrating a clear mortality benefit. Nevertheless, differences in study design must be acknowledged. Randomized trials typically employ strict inclusion criteria and protocol-driven interventions, whereas our registry reflects real-world practice with broader patient heterogeneity and operator-dependent decision-making. Therefore, our findings complement rather than substitute randomized evidence, providing external validity to current clinical practice.

### 4.3. Clinical Implications

Overall, these findings argue against a hierarchical approach to device selection and instead support individualized, patient-centered decision-making within multidisciplinary Pulmonary Embolism Response Teams (PERTs). While awaiting results from adequately powered randomized trials directly comparing catheter-based reperfusion strategies, our study provides robust real-world evidence indicating that USAT and MT offer comparable short-term safety and efficacy when applied in contemporary clinical practice. Taken together, these findings support a patient-centered, individualized approach to reperfusion strategy selection within multidisciplinary PERT frameworks. Rather than suggesting superiority of one technique over another, our data indicates that both USAT and MT are effective and safe options when appropriately selected. Treatment decisions should therefore continue to be guided by clinical presentation, bleeding risk, anatomical considerations, and institutional expertise.

### 4.4. Limitations

Several limitations of this study should be acknowledged. First, the observational and non-randomized design introduces the possibility of residual confounding, despite the use of PSM to balance baseline characteristics. We cannot exclude that treatment allocation may have been partially influenced by unmeasured factors, such as subtle hemodynamic instability; for example, serum lactate levels, a recognized marker of occult hypoperfusion, which was available for the larger part but not for all patients. It is therefore conceivable that the preferential selection of MT in patients with higher comorbidity burden also reflected perceived early hemodynamic vulnerability. Second, the relatively modest sample size, particularly in the MT cohort, limits statistical power to detect small but clinically meaningful differences in infrequent outcomes, including major bleeding or PE-related mortality. Treatment allocation was not randomized and was determined by multidisciplinary PERTs according to local protocols and physician judgment. Therefore, despite the use of PSM to balance measured baseline characteristics, the possibility of residual confounding and indication bias remains inherent to the study design. Unmeasured or incompletely captured factors influencing treatment selection, such as subtle differences in clinical severity, perceived bleeding risk, anatomical considerations, or operator preference, may have affected the choice between ultrasound-assisted catheter-directed thrombolysis and mechanical thrombectomy. This inherent degree of clinical discretion may have introduced selection bias and limits the reproducibility of our findings across different healthcare settings with varying levels of expertise, organizational structures, and procedural preferences. Consequently, although our findings reflect real-world practice and may enhance external validity, no significant differences in short-term outcomes were observed between the two strategies; however, these results should be interpreted as exploratory and hypothesis-generating rather than definitive, and external validation in other real-world cohorts and, ideally, randomized studies is required to confirm their generalizability. Moreover, echocardiographic assessment was restricted to the early post-procedural period, preventing evaluation of longer-term RV recovery, functional status, or the development of chronic thromboembolic pulmonary hypertension. Additionally, follow-up was limited to short-term outcomes, and the study reflects practice patterns from high-volume tertiary centers in Italy, which may limit generalizability to other settings or patient populations. Although no statistically significant differences were observed between USAT and MT after propensity score matching, some numerical trends toward higher cardiovascular and PE-related mortality, as well as increased bleeding rates in the USAT group, were noted. These findings should be interpreted with caution. First, the relatively small sample size and low event rates limit the statistical power of the analysis to detect modest but potentially clinically relevant differences between treatment strategies. Second, despite adjustment, residual confounding related to treatment selection cannot be excluded. Finally, differences in the underlying mechanisms of action, local thrombolysis versus mechanical clot extraction, may differentially influence bleeding risk and early clinical outcomes. Therefore, our results should be considered hypothesis-generating and warrant confirmation in adequately powered randomized trials. Despite these limitations, the registry provides valuable real-world evidence supporting the short-term safety and efficacy of USAT and MT in intermediate-high-risk PE.

## 5. Conclusions

In conclusion, in this real-world multicenter registry of intermediate-high-risk PE, both USAT and MT were associated with rapid improvements in hemodynamic, RV function, and short-term clinical outcomes. These findings suggest that early reduction in RV afterload, rather than the specific device, drives clinical stabilization. Until results from randomized trials are available, USAT and MT appear to be safe and effective, supporting individualized, patient-centered selection within multidisciplinary PE response frameworks.

## Figures and Tables

**Table 1:** General characteristics of the baseline population. BMI: body mass index; CKD: chronic kidney dis ease; COPD: chronic obstructive pulmonary disease; CTPA: computed tomography pulmonary angiography; DOAC: direct oral anticoagulant; DVT: deep vein thrombosis; LV: left ventricle; PE: pulmonary embolism; DBP: diastolic blood pressure, HR: heart rate; HT: hypertension; LMWH: low molecular weight heparin; PASP: pulmonary artery systolic pressure; RV: right ventricle; SBP: systolic blood pressure; VKA: vitamin K antagonists. * Including FlowTriever^®^ and Indigo^®^. ** Data available in 191 patients (90.5%) treated with USAT and 73 patients (97.3%) treated with thrombectomy.

	USAT (n = 211)	Thrombectomy * (n = 75)	*p*
Age, years	64.7 ± 14.5	64.4 ± 17.8	0.88
Females, n (%)	111 (52.6)	39 (52.0)	0.92
BMI, kg/m^2^	29.6 ± 5.6	29.8 ± 5.9	0.78
SBP, mmHg	131.7 ± 11.4	125.9 ± 15.0	<0.001
DBP, mmHg	80.2 ± 13.3	75.2 ± 14.4	0.007
HR, bpm	101.6 ± 8.2	100.0 ± 6.4	0.12
HT, n (%)	108 (5.1)	44 (58.6)	0.26
Smokers			
Previous, n (%)	12 (10.9)	25 (33.3)	0.009
Active, n (%)	20 (9.4)	10 (13.3)	0.34
CKD, n (%)	21 (9.9)	22 (29.3)	<0.001
Diabetes Mellitus, n (%)	25 (11.8)	31 (41.3)	<0.001
COPD, n (%)	9 (4.3)	16 (21.3)	<0.001
Cancer, n (%)	9 (4.3)	16 (21.3)	0.56
Heart Failure, n (%)	10 (4.7)	3 (4.0)	<0.001
Coronary artery disease, n (%)	10 (4.7)	7 (9.3)	0.14
Stroke, n (%)	8 (3.8)	6 (8.0)	0.13
Previous DVT, n (%)	28 (13.3)	15 (20.0)	0.16
Previous PE, n (%)	13 (6.2)	5 (6.6)	0.90
Echocardiography			
RV/LV ratio	1.2 ± 0.1	1.4 ± 0.2	<0.001
Hypokinesia, n (%)	161 (76.3)	32 (42.6)	<0.001
TAPSE, mm	15.8 ± 4.7	14.7 ± 3.6	0.04
PASP, mmHg	51.0 ± 16.2	51.4 ± 12.4	0.84
CTPA			
Bilateral, main, n (%)	149 (70.6)	47 (62.6)	0.20
Bilateral, lobar, n (%)	44 (20.8)	21 (28.0)	0.21
Unilateral, main, n (%)	15 (7.1)	6 (8.0)	0.79
Unilateral, lobar, n (%)	3 (1.4)	1 (1.3)	0.94
Baseline serum lactate level (mmol/L) **	1.6 [1–2–2.11]	2.3 [1–7–3.1]	<0.001
Device for Thrombectomy, n (%)			
FlowTriever^®^, n (%)	-	31 (45)	-
Indigo^®^, n (%)	-	38 (55)	-
Anticoagulant treatment			
DOAC, n (%)	132 (62.5)	53 (70.6)	0.20
LMWH, n (%)	63 (29.8)	21 (28.0)	0.76
VKA, n (%)	16 (7.5)	1 (1.3)	0.06

**Table 2:** General characteristics and outcomes of the population analyzed after 1:1 propensity score matching, using a caliper of 0.10. SMD values close to 0 indicate better covariate balance between groups, with values < 0.10 generally considered indicative of adequate balance, 0.10–0.20 of small imbalance, and >0.20 of meaningful imbalance. Cause-specific mortality categories (cardiovascular, non-cardiovascular, and PE-related death) were adjudicated independently and are not mutually exclusive; therefore, their numerical totals should not be interpreted as additive or directly comparable to all-cause mortality BMI: body mass index; CKD: chronic kidney disease; COPD: chronic obstructive pulmonary disease; CTPA: computed tomography pulmonary angiography; DOAC: direct oral anticoagulant; DVT: deep vein thrombosis; LV: left ventricle; PE: pulmonary embolism; DBP: diastolic blood pressure, HR: heart rate; HT: hypertension; LMWH: low molecular weight heparin; PASP: pulmonary artery systolic pressure; RV: right ventricle; SBP: systolic blood pressure; VKA: vitamin K antagonists. * Including FlowTriever and Cat Penumbra.

	USAT (n = 69)	Thrombectomy * (n = 69)	*p*	SMD
Age, years	63.7 ± 11.7	63.6 ± 13.4	0.95	0.01
Females, n (%)	29 (42.0)	31 (44.4)	0.77	0.05
BMI, kg/m^2^	29.2 ± 3.4	29.1 ± 5.0	0.84	0.02
SBP, mmHg	121.9 ± 13.4	120.1 ± 14.7	0.33	0.09
DBP, mmHg	73.5 ± 13.1	72.1 ± 12.8	0.42	0.08
HR, bpm	100.4 ± 8.9	99.9 ± 7.2	0.66	0.06
HT, n (%)	29 (42.0)	32 (46.3)	0.22	0.09
Smokers				
Previous, n (%)	9 (13.0)	13 (18.8)	0.35	0.10
Active, n (%)	10 (14.4)	7 (10.1)	0.44	0.08
CKD, n (%)	16 (23.1)	19 (27.5)	0.55	0.09
Diabetes Mellitus, n (%)	21 (30.4)	24 (34.7)	0.59	0.09
COPD, n (%)	7 (10.1)	12 (17.3)	0.22	0.03
Cancer, n (%)	15 (21.7)	10 (14.4)	0.26	0.06
Heart Failure, n (%)	8 (11.5)	2 (2.8)	0.06	0.08
Coronary artery disease, n (%)	8 (11.5)	5 (7.2)	0.38	0.09
Stroke, n (%)	6 (8.6)	4 (5.7)	0.51	0.08
Previous DVT, n (%)	18 (26.0)	12 (17.3)	0.21	0.06
Previous PE, n (%)	7 (10.1)	3 (4.3)	0.45	0.08
Echocardiography				
RV/LV ratio	1.2 ± 0.3	1.3 ± 0.4	0.09	0.08
Hypokinesia, n (%)	30 (43.4)	28 (40.5)	0.73	0.05
TAPSE, mm	15.2 ± 1.4	14.3 ± 1.1		0.07
PASP, mmHg	49.3 ± 11.3	50.3 ± 10.6	0.18	0.09
CTPA				
Bilateral, main, n (%)	37 (54.4)	44 (63.7)	0.26	0.09
Bilateral, lobar, n (%)	21 (30.4)	20 (28.9)	0.84	0.09
Unilateral, main, n (%)	9 (13.0)	4 (5.7)	0.14	0.08
Unilateral, lobar, n (%)	2 (2.8)	1 (1.4)	0.56	0.06
Baseline serum lactate level (mmol/L)	1.9 [1.4–2.4]	2.1 [1.5–2.7]	0.12	0.10
Anticoagulant treatment				
DOAC, n (%)	45 (65.2)	47 (68.1)	0.71	-
LMWH, n (%)	18 (26.0)	21 (30.4)	0.56	-
VKA, n (%)	6 (8.6)	1 (1.4)	0.06	-
Inferior vena cava filter, n (%)	4 (5.7)	10 (14.4)	0.10	-
Blood Transfusion, n (%)	7 (10.1)	6 (8.6)	0.76	-
Outcomes				
Death, all-causes, n (%)	9 (13)	8 (11.5)	0.78	
Cardiovascular death, n (%)	6 (8.6)	1 (1.4)	0.58	
Non-Cardiovascular death, n (%)	7 (10.1)	8 (11.5)	0.79	
PE-related, n (%)	4 (5.7)	1 (1.4)	0.17	

**Table 3:** Between-group comparison of echocardiographic changes (Δ values) in the propensity score–matched cohort. Values are expressed as mean ± standard deviation (SD). Δ values represent absolute change from baseline to post-procedural echocardiographic assessment with a relative 95% confidence interval. Between-group comparisons were performed using an independent samples *t*-test for normally distributed continuous variables (Δ RV/LV ratio, Δ TAPSE, Δ PASP), and normality was assessed visually and by the Shapiro–Wilk test. USAT: ultrasound-assisted catheter-directed thrombolysis; MT: mechanical thrombectomy; RV: right ventricle; LV: left ventricle; TAPSE: tricuspid annular plane systolic excursion; PASP: pulmonary artery systolic pressure.

Echocardiographic Parameter	USAT (n = 69)	MT (n = 69)	Median Difference [95% CI] (USAT vs. MT)	*p* Value
Δ RV/LV ratio	−0.41 ± 0.18	−0.38 ± 0.20	−0.03 [−0.10 to 0.04]	0.42
Δ TAPSE (mm)	+7.4 ± 2.1	+6.9 ± 2.4	+0.50 [−0.27 to 1.27]	0.31
Δ PASP (mmHg)	−18.6 ± 6.3	−16.7 ± 7.1	−1.90 [−4.22 to 0.42]	0.18

## Data Availability

Data are available, upon reasonable request, by contacting the corresponding author.

## Supplementary Materials

The following supporting information can be downloaded at: https://www.mdpi.com/article/10.3390/jcm15114023/s1, Supplementary Table S1: List of enrolling centres of the USAT IH-PE Registry; Supplementary Table S2: General characteristics of the baseline population treated with mechanical thrombectomy after propensity score matching; Supplementary Table S3: Changes of transthoracic echocardiographic indices in the USAT cohort after propensity score matching; Supplementary Table S4: Changes of transthoracic echocardiographic indices in the thrombectomy cohort after propensity score matching; Supplementary Table S5: Changes of transthoracic echocardiographic indices in the mechanical thrombectomy cohort, stratified by device, in the propensity score matched cohort. Supplementary Table S6: Log-rank (Mantel-Cox) analysis for different outcomes between patients treated with FlowTriever^®^ of Indigo^®^ system; Supplementary Table S7: Bleeding events according to the Bleeding Academic Research Consortium (BARC) classification after propensity score matching.

## Abbreviations

The following abbreviations are used in this manuscript:

BMI	Body mass index
CDT	Catheter-directed therapy
CKD	Chronic kidney disease
COPD	Chronic obstructive pulmonary disease
CTPA	Computed tomography pulmonary angiography
CV	Cardiovascular
DBP	Diastolic blood pressure
DOAC	Direct oral anticoagulant
DVT	Deep vein thrombosis
ESC	European Society of Cardiology
LMWH	Low molecular weight heparin
LV	Left ventricle
MT	Mechanical thrombectomy
PASP	Pulmonary artery systolic pressure
PE	Pulmonary Embolism
PERT	Pulmonary Embolism Response Team
PSM	Propensity score matching
RAP	Right atrial pressure
RV	Right ventricle
RVD	Right ventricular dysfunction
SBP	Systolic blood pressure
TAPSE	Tricuspid annular plane systolic excursion
TTE	Transthoracic echocardiography
UFH	Unfractionated heparin
USAT	Ultrasound-assisted catheter-directed thrombolysis
VKA	Vitamin K antagonist
