# Supplementary material for: Comparative Outcomes of Ultrasound-Assisted Thrombolysis and Mechanical Thrombectomy in Intermediate-High-Risk Pulmonary Embolism
Source: J Clin Med. 2026 May 22;15(11):4023. doi: 10.3390/jcm15114023 (PMC13258309; doi:10.3390/jcm15114023)
Supplement: Supplementary file 1 [file jcm-15-04023-s001.zip › jcm-4296358-supplementary.pdf]

Comparative Outcomes of Ultrasound-Assisted Thrombolysis and Mechanical Thrombectomy in Intermediate-High-Risk Pulmonary Embolism

Claudia Colombo [1,*], Marco Zuin [2,3,4,*], Filippo Russo [5], Mario Iannaccone [6], Marco Solcia [7], Francesco Musca [8], Ilaria Emanuela Bossi [9], Andrea Cesari [1], Elena Gualini [1], Federica Fumarola [10], Alberto Balderi [11], Francesca Giordana [12], Andrea Discalzi [13], Lorenzo Tua [14], Stefano Buratti [15], Ruggero Vercelli [16], Lorenzo Cianfanelli [17], Marianna Adamo [18], Alaide Chieffo [5], Giacomo Bocuzzi [6], Matteo Montorfano [5,19], Fabrizio Oliva [1] and Alice Sacco [1,§]

** CC and MZ contributed equally to this work and share first authorship.

1 Cardiology 1, De Gasperis Cardio Center, ASST Grande Ospedale Metropolitano Niguarda, Milan, Italy

2 Department of Translational Medicine, University of Ferrara, Ferrara, Italy;

3 Department of Cardio-Thoraco-Vascular Sciences and Public Health, University of Padova, Padua, Italy

4 Department of Cardiology, Madre Teresa di Calcutta Hospital, AULSS 6, South Padova Hospitals, Schiavonia, Italy.

5 Interventional Cardiology Unit, IRCCS San Raffaele Scientific Institute, Milan, Italy

6 Cardiology Unit San Giovanni Bosco, ASL Città di Torino, Turin, Italy

7 Interventional Radiology Unit, ASST Grande Ospedale Metropolitano Niguarda, Milan, Italy

8 Cardiology 4, De Gasperis Cardio Center, ASST Grande Ospedale Metropolitano Niguarda, Milan, Italy

9 Department of Emergency Medicine, ASST Grande Ospedale Metropolitano Niguarda, Milan, Italy

10 Interventional Radiology Unit, San Giovanni Bosco, ASL Città di Torino, Turin, Italy

11 Unit of Interventional Radiology, Department of Radiology, A.O. S. Croce e Carle, Cuneo, Italy

12 Division of Cardiology, A.O. S. Croce e Carle, Cuneo, Italy

13 Radiology Unit, Department of Surgical Sciences, University of Torino, Turin, Italy

14 Interventional Cardiology Unit, ASST Santi Paolo e Carlo, San Carlo Borromeo Hospital, Milan, Italy

15 Cardiology Division, ASST Santi Paolo and Carlo, San Paolo Hospital, Milan, Italy

16Interventional Radiology Unit, ASST Santi Paolo Carlo, Milan, Italy

17 Intensive Care Unit, IRCCS San Raffaele Scientific Institute, Milan, Italy

18 Institute of Cardiology, ASST Spedali Civili di Brescia, Brescia, Italy

19 School of Medicine, Vita-Salute San Raffaele University, Milan, Italy and Interventional Cardiology Unit IRCCS San Raffaele Scientific Institute, Milan, Italy.

Supplementary Files

Supplementary Table S1. List of enrolling centres of the USAT IH-PE Registry. 3

Supplementary Table S2. General characteristics of the baseline population treated with mechanical thrombectomy after propensity score matching.. 5

Supplementary Table S3. Changes of transthoracic echocardiographic indices in the USAT cohort after propensity score matching. 6

Supplementary Table S4. Changes of transthoracic echocardiographic indices in the thrombectomy cohort after propensity score matching.. 7

Supplementary Table S5. Changes of transthoracic echocardiographic indices in the mechanical thrombectomy cohort, stratified by device, in the propensity score matched cohort. 8

Supplementary Table S6. Log-rank (Mantel-Cox) analysis for different outcomes between patients treated with FlowTriever® of Indigo® system.. 9

Supplementary Table S7. Bleeding events according to the Bleeding Academic Research Consortium (BARC) classification after propensity score matching. 10

**Enrolling Centre**
ASST Niguarda, Milan
Ospedale San Giovanni Bosco, Turin

Ospedale S. Croce e Carle, Cuneo
IRCCS Ospedale San Raffaele, Milan
Ospedale Le Molinette, Turin
ASST degli Spedali Civili, Brescia
Ospedale San Carlo, Milan
Ospedale San Paolo, Milan

Supplementary Table S1. List of enrolling centres of the USAT IH-PE Registry.

	**FlowTriever®**	**Indigo®**	**p**

	(n=31)	(n=38)	
Age, years	64.1±12.9	63.2±13.8	0.78
Females, n (%)	14 (45.2)	17 (44.7)	0.96
BMI, Kg/m²	29.3±4.6	29.0±5.3	0.81
SBP, mmHg	119.8±14.2	120.4±15.1	0.87
DBP, mmHg	71.9±12.4	72.3±13.1	0.90
HR, bpm	99.4±7.5	100.2±7.1	0.63
HT, n (%)	14 (45.2)	18 (47.4)	0.85
CKD, n (%)	8 (25.8)	11 (28.9)	0.77
Diabetes Mellitus, n (%)	10 (32.3)	14 (36.8)	0.69
COPD, n (%)	5 (16.1)	7 (18.4)	0.80
Cancer, n (%)	4 (12.9)	6 (15.8)	0.74
RV/LV ratio	1.31±0.4	1.29±0.3	0.82
TAPSE, mm	14.4±1.2	14.2±1.1	0.52
PASP, mmHg	49.8±9.9	50.7±11.2	0.71
Bilateral main PE, n (%)	20 (64.5)	24 (63.2)	0.91
Bilateral lobar, n (%)	7 (22.6)	9 (23.7)	0.92
Unilateral main, n (%)	3 (9.7)	1 (1.6)	0.18
Unilateral lobar, n(%)	1 (3.2)	4 (10.5)	0.22
Baseline serum lactate (mmlo/L)	2.0 [1.5-2.6]	2.0 [1.6-2.8]	0.39
Anticocoagulation treatment			
DOAC, n (%)	21 (67.7)	26 (68.4)	0.95
LMWH, N (%)	9 (29.0)	12 (31.6)	0.81
VKA, n (%)	1 (3.2)	0	0.27
Inferior vena cava filter, n (%)	4 (12.9)	6 (15.8)	0.74
All-cause of death, n (%)	3 (9.7)	3 (9.7)	1.00
Cardiovascular death, n (%)	1 (3.2)	0	0.31
PE-related death, n (%)	1 (3.2)	0	0.31
Blood transfusion, n (%)	2 (6.5)	2 (6.5)	1.00

Any BARC bleeding, n (%)	5 (16.1)	4 (12.9)	0.72

Supplementary Table S2. General characteristics of the baseline population treated with mechanical thrombectomy after propensity score matching. BMI: Body mass index; CKD: Chronic kidney disease; COPD: Chronic obstructive pulmonary disease; CTPA: Computed tomography pulmonary angiography: DOAC: Direct oral anticoagulant; DVT: Deep vein thrombosis; LV: Left ventricle; PE: Pulmonary embolism; DBP: Diastolic blood pressure, HR: Heart rate; HT: Hypertension; LMWH: Low molecular weight heparin; PASP: Pulmonary artery systolic pressure; RV: Right ventricle; SBP: Systolic blood pressure; VKA: Vitamin K antagonists.

Outcomes - USAT	Before the procedure n=69	After the procedure N=69	p
RV/LV	1.2±0.3	0.79±0.1	<0.001
TAPSE, mm	15.2±1.4	22.6±0.3	<0.001
PAPs, mmHg	49.3±3.1	30.7±3.2	<0.001

Supplementary Table S3. Changes of transthoracic echocardiographic indices in the USAT cohort after propensity score matching. LV: Left ventricle; PASP: Pulmonary artery systolic pressure; TAPSE: Tricuspid Annular Plane Systolic Excursion.

Outcomes - thrombectomy	Before the procedure n=69	After the procedure n=69	p
RV/LV	1.3±0.4	0.90±0.2	<0.001
TAPSE, mm	14.3±1.1	18.9±0.8	<0.001
PAPs, mmHg	50.3±10.6	33.6±4.2	<0.001

Supplementary Table S4. Changes of transthoracic echocardiographic indices in the thrombectomy cohort after propensity score matching. LV: Left ventricle; PASP: Pulmonary artery systolic pressure; TAPSE: Tricuspid Annular Plane Systolic Excursion.

	**Before**<br>**N=31**	**After**<br>**N=31**	**p**
**FlowTriever®**			
RV/LV	1.30±0.3	0.88±0.2	<0.001
TAPSE, mm	14.3±1.2	19.2±0.9	<0.001
PASP, mmHg	50.0±10.2	33.0±4.0	<0.001
**Indigo®**			
RV/LV	1.29±0.3	0.91±0.2	<0.001
TAPSE, mm	14.3±1.1	18.7±0.7	<0.001
PASP, mmHg	50.1±10.5	34.1±4.4	<0.001

Supplementary Table S5. Changes of transthoracic echocardiographic indices in the mechanical thrombectomy cohort, stratified by device, in the propensity score matched cohort. LV: Left ventricle; PASP: Pulmonary artery systolic pressure; TAPSE: Tricuspid Annular Plane Systolic Excursion.

**Outcomes**	**FlowTriever®** **N=31**	**Indigo®** N=31	**Log-rank Mantel–Cox**
All-cause of death, n (%)	3 (9.7)	2 (6.4)	0.98
CV death, n (%)	1 (3.2)	0	0.88
Non-CV death, n (%)	1 (3.2)	0	0.88
PE-related death, n (%)	0	1 (3.2)	0.86

Supplementary Table S6. Log-rank (Mantel-Cox) analysis for different outcomes between patients treated with FlowTriever® of Indigo® system. CV: Cardiovascular; PE: Pulmonary embolism.

	**USAT (n=69)**	**Thrombectomy* (n=69)**	**p**
**No bleeding**	47 (68.1)	56 (81.1)	0.08
BARC 0, n (%)	10 (14.4)	7 (10.1)	0.44
BARC 1, n (%)	4 (5.7)	6 (8.6)	0.51
BARC 2, n (%)	2 (2.8)	0	0.18
BARC 3a, n, (%)	3 (4.3)	0	0.16
BARC 3b, n (%)	1 (1.4)	0	0.37
BARC 3c, n (%)	1 (1.4)	0	0.37
BARC 4, n (%)	1 (1.4)	0	0.37

Supplementary Table S7. Bleeding events according to the Bleeding Academic Research Consortium (BARC) classification after propensity score matching. * Including FlowTriever and Cat Penumbra.
